# Bacterial and Bacteriophage Antibiotic Resistance in Marine Bathing Waters in Relation to Rivers and Urban Streams

**DOI:** 10.3389/fmicb.2021.718234

**Published:** 2021-07-26

**Authors:** Laura Sala-Comorera, Tristan M. Nolan, Liam J. Reynolds, Anjan Venkatesh, Lily Cheung, Niamh A. Martin, Jayne H. Stephens, Aurora Gitto, Gregory M. P. O’Hare, John J. O’Sullivan, Wim G. Meijer

**Affiliations:** ^1^UCD School of Biomolecular and Biomedical Science, UCD Earth Institute, UCD Conway Institute, University College Dublin, Dublin, Ireland; ^2^UCD School of Computer Science, UCD Earth Institute, University College Dublin, Dublin, Ireland; ^3^UCD School of Civil Engineering, UCD Dooge Centre for Water Resources Research, UCD Earth Institute, University College Dublin, Dublin, Ireland

**Keywords:** antibiotic resistance genes, microbial source tracking, bacteriophages, fecal pollution, rivers, urban streams, bathing waters

## Abstract

Fecal pollution of surface water may introduce bacteria and bacteriophages harboring antibiotic resistance genes (ARGs) into the aquatic environment. Watercourses discharging into the marine environment, especially close to designated bathing waters, may expose recreational users to fecal pollution and therefore may increase the likelihood that they will be exposed to ARGs. This study compares the bacterial and bacteriophage ARG profiles of two rivers (River Tolka and Liffey) and two small urban streams (Elm Park and Trimleston Streams) that discharge close to two marine bathing waters in Dublin Bay. Despite the potential differences in pollution pressures experienced by these waterways, microbial source tracking analysis showed that the main source of pollution in both rivers and streams in the urban environment is human contamination. All ARGs included in this study, *bla*_*TEM*_, *bla*_*SHV*_, *qnrS*, and *sul1*, were present in all four waterways in both the bacterial and bacteriophage fractions, displaying a similar ARG profile. We show that nearshore marine bathing waters are strongly influenced by urban rivers and streams discharging into these, since they shared a similar ARG profile. In comparison to rivers and streams, the levels of bacterial ARGs were significantly reduced in the marine environment. In contrast, the bacteriophage ARG levels in freshwater and the marine were not significantly different. Nearshore marine bathing waters could therefore be a potential reservoir of bacteriophages carrying ARGs. In addition to being considered potential additional fecal indicators organism, bacteriophages may also be viewed as indicators of the spread of antimicrobial resistance.

## Introduction

Microbial antibiotic resistance is a severe threat to public health, resulting in failure to treat a range of infections, in extended hospital treatment and in increased healthcare costs (Stewardson et al., 2016; Cassini et al., 2019; Roope et al., 2019; Jit et al., 2020). Furthermore, it is estimated that multidrug resistant pathogens will lead to 10 million deaths by 2050 (OECD, 2018; WHO, 2019). Initially, the problem was mainly approached from a clinical perspective. However, more recently, the importance of the environment as a major contributor to the spread of antimicrobial resistance in the human and animal population has become increasingly clear (Allcock et al., 2017; Leonard et al., 2018; Hernando-Amado et al., 2020). Successful management strategies and policies to combat the increase in antimicrobial resistance will therefore have to be based on a One Health approach, which recognizes the connectivity between animal, human, and environmental health (Hernando-Amado et al., 2019; Van Bruggen et al., 2019).

Antibiotic resistance in bacteria may arise from chromosomal mutations, however, for most types of antimicrobial resistance, the acquisition of antibiotic resistance genes (ARGs) mediated by horizontal gene transfer mechanisms is a more common mechanism (Andersson and Hughes, 2010; Huddleston, 2014; Baquero et al., 2019). Transduction by bacteriophages is an important mechanism in spreading ARGs within a microbial population (Muniesa et al., 2013b; Balcazar, 2014; Brown-Jaque et al., 2015; Balcázar, 2018; Maganha De et al., 2021). The importance of bacteriophages in spreading ARGs and virulence genes, was recently underscored by the discovery of lateral transduction, which is the basis of genomic hypermobility (Chen et al., 2018; Chiang et al., 2019).

Environments characterized by high levels of fecal matter such as sewage, animal slurry, sludge, and effluent of wastewater treatment plants are hotspots of antibiotic resistant bacteria and phages harboring ARGs, forming an ideal environment for horizontal gene transfer to occur (Calero-Cáceres et al., 2014; Quirós et al., 2014; Ross and Topp, 2015; Calero-Cáceres and Muniesa, 2016; Guo et al., 2017; Yang et al., 2021; Zieliński et al., 2021). It has been shown that transfection and transduction of ARGs from environmental phages conferred resistance to the recipient bacteria (Battaglioli et al., 2011; Colomer-Lluch et al., 2011; Gunathilaka et al., 2017; Wang et al., 2018b; Yang et al., 2021).

The effluent of wastewater treatment plants is an important route by which ARGs may enter the aquatic environment (Rizzo et al., 2013; Marti et al., 2014b; Rodriguez-Mozaz et al., 2015; Lekunberri et al., 2017; Zhou et al., 2020; Nguyen et al., 2021). Sewerage misconnections and leaking septic tanks may be a source of untreated sewage entering rivers and other waterbodies (Kay et al., 2008; Hinojosa et al., 2020; Reynolds et al., 2021). Furthermore, agricultural land run-off may also negatively impact water quality and introduce ARGs into waterbodies (Unc and Goss, 2004; Ballesté et al., 2020). Watercourses discharging into the marine environment, especially close to designated bathing waters, may expose the users to fecal pollution and therefore may increase the likelihood that they will be exposed to ARGs (Molina et al., 2014; Leonard et al., 2018; Ahmed et al., 2020; Reynolds et al., 2020; Sala-Comorera et al., 2021b).

This study focuses on the bacterial and bacteriophage ARG profiles of two rivers entering a large urban environment and two small, completely urban, streams that discharge close to two marine bathing waters. We show that the ARG profiles of these streams and rivers in an urban environment are highly similar and have a strong impact on nearshore marine bathing waters. In contrast to bacterial ARGs, ARGs associated with bacteriophages appear to persist in the nearshore marine environment.

## Materials and Methods

### Site Location

Dublin, the capital of Ireland, is a coastal city on the Irish Sea with 560,000 inhabitants. Near 1,905,000 people live within the greater Dublin area and around sixty watercourses, ranging from major rivers to small streams, which discharge into Dublin Bay. Dublin Bay, which is a UNESCO biosphere, has three designated bathing waters: Dollymount, Sandymount, and Merrion Strands.

**FIGURE 1:**
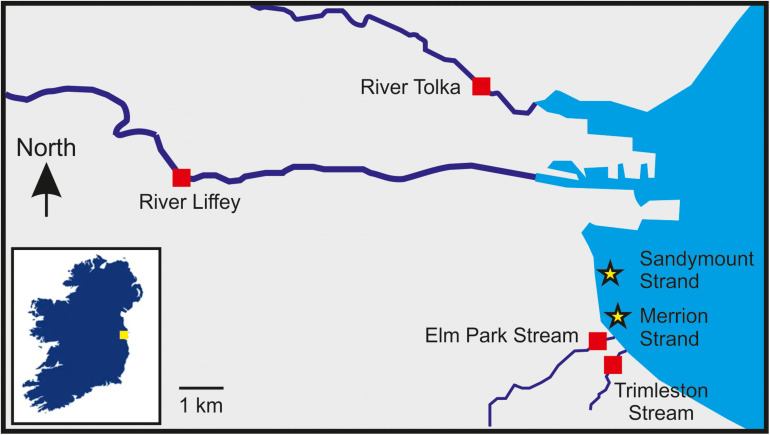
Location of the sampling sites selected in this study. The sampling stations are indicated by red squares (rivers and streams) and stars (bathing waters). The inset box shows the location of Dublin Bay in Ireland indicated by a yellow square.

In this study, two rivers (River Liffey and River Tolka), two streams (Elm Park Stream and Trimleston Stream), and two bathing waters (Merrion Strand and Sandymount Strand) were selected (Figure 1 and Supplementary Table 1). The River Liffey rises in a pristine area near Kippure in the Wicklow Mountains and flows through agricultural land to reaching Dublin city. The River Liffey has a 125 km course and it is the main river flowing into Dublin Bay (Sweeney, 2017). The River Tolka is 33 km long, and it is the second largest river by flow in Dublin Bay. The river rises near Culmullin Cross Road and flows through agricultural and industrial land into the north of Dublin city (Sweeney, 2017). The Elm Park Stream and the Trimleston Stream catchment is completely urban areas with a population of 40,000 people. The length of the streams are 3.8 and 1.7 km, respectively, and the depth is less than 10 cm. Both streams discharge close to a designated bathing area in Dublin Bay.

Grab samples were collected at the tidal limit at a depth of 10–20 cm, every 5 weeks over 15 months (from September 2018 to November 2019). Bathing water samples were taken during high tide at Merrion and Sandymount Strands. A total of 85 samples collected and stored at 4°C before being processed within 6 h.

### Enumeration of Fecal Indicator Organisms

The levels of *Escherichia coli* and intestinal enterococci were determined by membrane filtration. Water samples were filtered through 0.45 μm pore size nitrocellulose membranes (Thermo Scientific) and placed on Tryptone Bile X-Glucuronide agar (Sigma-Aldrich) at 37°C for 4 h, followed by an incubation at 44°C for 18 h to enumerate *E. coli* (ISO, 2001). Intestinal enterococci were enumerated by incubating the membrane on Slanetz and Bartley agar (Oxoid) at 37°C for 48 h. After incubation, membranes were transferred into Bile Aesculin agar at 44°C for 2 h to confirm positive intestinal enterococci colonies (ISO, 2000).

### Electron Microscopy

River samples (100 ml) were concentrated by ultrafiltration using 100 kDa Amicon Ultra-15 Centrifugal Filter units (Millipore) and 5 μl of the concentrated samples were pipetted onto a 200-mesh copper grid coated with formvar. Samples were negatively stained with 5 μl of 2% uranyl acetate stain and incubated for 2 min. The grids were imaged using a transmission electron microscope Tecnai G2 (FEI Tecnai) operating at 120 kV. Untreated sewage (50 ml) collected in the influent of a local wastewater treatment plant was used as a positive control.

### DNA Extraction From the Bacterial Fraction

DNA of the water samples was extracted after concentrating by filtering 100 ml through 0.22 μm mixed cellulose ester membrane filters. The filters were then transferred in 500 μl of GITC buffer [5 M guanidine thiocyanate, 100 mM EDTA (pH 8), and 0.5% sarkosyl] and stored at −20°C. DNA was extracted using the DNeasy Blood and Tissue kit (Qiagen) with some modifications as reported previously (Gourmelon et al., 2007). The DNA was eluted in a final volume of 70 μl.

### DNA Extraction From the Bacteriophage Fraction

DNA of bacteriophages present in water was extracted using the protocol described by Colomer-Lluch et al. (2011). Briefly, 100 ml of water was passed through 0.22 μm low protein binding polyethersulfone filters. The filtrate was concentrated 200-fold using 100 kDa Amicon Ultra-15 Centrifugal Filter units (Millipore), and subjected to chloroform extraction in a 1:1 (v/v) ratio, followed by a DNAse (100 U/ml) treatment at 37°C for 1 h and 10 min at 80°C. At this point, an aliquot of 10 μl was collected as a control to ensure the complete removal of free DNA. Phage particles were subjected to proteinase K digestion (0.2 mg/ml) for 1 h at 56°C followed by phenol/chloroform extraction and ethanol precipitation. The resulting DNA was dissolved into 20 μl water.

### Quantification of Gene Target

Primers to amplify the ARGs *bla*_*TEM*_, *bla*_*SHV*_, *qnrS*, and *sul1* and the human (HF183) and ruminant (CF128) microbial source tracking markers, as well as the cycling conditions are described in Supplementary Table 2. The MST markers and ARGs were quantified as previously described (Ballesté et al., 2020; Reynolds et al., 2020). Standard curves were generated using linearized cloned standards between 10^0^ and 10^6^ gene copies to quantify target gene levels in each sample (Ballesté et al., 2020; Reynolds et al., 2020). The limit of detection of each assay was determined as the lowest concentration of DNA target detected in 95% or more of replicates, whereas the limit of quantification was determined as the lowest concentration of DNA quantified within 0.5 SD of the log_10_ concentration (Supplementary Table 2; Blanchard et al., 2012; AFNOR, 2015). All samples and negative controls were analyzed in duplicate in each 96-well plate. The absence of non-packaged DNA in the bacteriophage extraction protocol was verified with 16S rRNA gene amplification by PCR (AllTaq Master Mix Kit, Qiagen) and ARGs genes by qPCR. Only negative samples were used for the subsequent analysis. MST markers and ARGs concentrations were expressed as gene copies per 100 ml (GC/100 ml). The amplification efficiency of each reaction was calculated using the *E* = 10^(1/slope)^ − 1 equation (Rutledge, 2003).

### Data Analysis

The non-parametric Mann–Whitney paired-test and Kruskal–Wallis test with Dunn’s *post hoc* analysis was used to assess significant differences between microbial source tracking markers and ARGs between waterbodies. The values of the qPCR targets were log_10_ transformed and Spearman correlation was used to identify relationships between variables. A significance cut-off of *p* ≤ 0.05 was used for all analyses. Statistical analysis was carried out using GraphPad Prism 9.1.0. software (GraphPad Software).

## Results

### Fecal Contamination of Rivers, Urban Streams, and Bathing Waters

**FIGURE 2:**
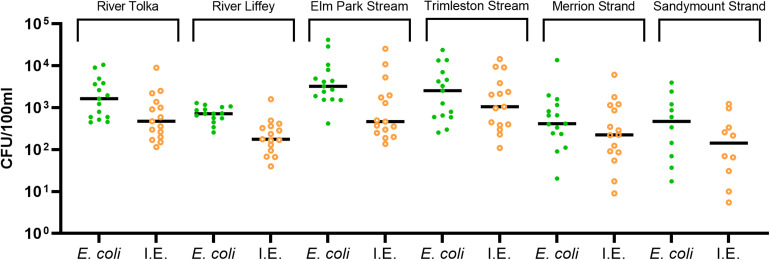
Levels of the fecal indicators *Escherichia coli*, intestinal enterococci (I.E.). The dots show the concentration of each sample and the median are indicated as a horizontal line.

All water samples (*n* = 85) were positive for *E. coli* and intestinal enterococci (Figure 2). The River Tolka and the River Liffey, which flow through agricultural areas before reaching the city, had similar median levels of fecal indicator organisms as the small urban Elm Park and Trimleston streams. The fecal indicators in the latter varied more than three orders of magnitude. In contrast, the fecal indicator levels were less variable for the River Liffey during this 15-month period, which had the lowest median concentration for the fecal indicators. In general, all rivers and streams received fecal contamination along their course, independently of the catchment area.

The median fecal indicator levels in Merrion and Sandymount Strands were similar with individual values varying by up to four orders of magnitude. Some of the bathing water samples analyzed exceeded the 90th percentile value for sufficient water quality parameters for coastal waters (≥500 CFU/100 ml for *E. coli* and ≥185 CFU/100 ml for intestinal enterococci) according to the European bathing water quality Directive (EU, 2006).

### Fecal Contamination of Rivers and Streams Is Predominantly Human in Nature

**FIGURE 3:**
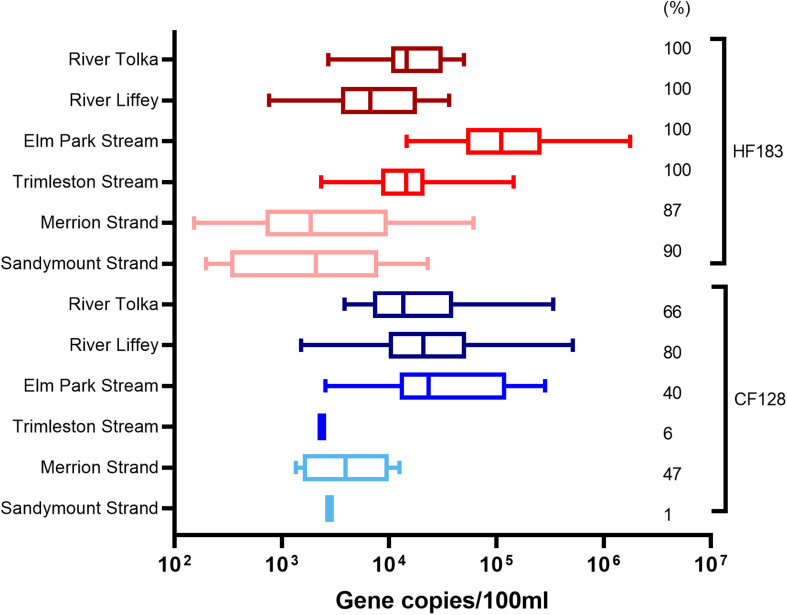
Boxplot representation of the concentrations and percentage of positive samples for the human (HF183) and ruminant (CF128) marker in water samples from rivers (Tolka and Liffey), streams (Elm Park and Trimleston), and bathing waters (Sandymount and Merrion Strands). In the boxplots the lower hinge represents 25% quantile, upper hinge 75% quantile, and center line the median. The whiskers show the maximum and the lowest value. The percentage of samples above the quantification limit is indicated, only values above the quantification limit are plotted.

The *Bacteroidales* human (HF183) and ruminant (CF128) markers were deployed to determine the biological origins of pollution (Figure 3). The River Liffey catchment is larger than that of the River Tolka and has more agricultural land use. Therefore, as expected, more River Liffey (80%) than River Tolka (66%) samples were positive for the ruminant marker. The levels of the ruminant marker reached values of 5.1 × 10^5^ and 3.4 × 10^5^ GC/100 ml in River Liffey and River Tolka, respectively. In contrast the marker was only sporadically detected in the Elm Park stream (40%) and in only one sample in the Trimleston stream. When the CF128 marker tested positive in Elm Park stream samples, concentrations up to 2.8 × 10^5^ GC/100 ml were reported. The presence of this marker in an urban stream is explained by the run-off from a small pasture grazed by a few heads of cattle on a private property near the Elm Park stream. Similar to the streams, the ruminant CF128 marker was present in 47% of the samples in Merrion Strand and in only one sample in Sandymount Strand.

The human marker was detected in all river and stream samples and in nearly 90% of the bathing water samples. HF183 levels in the streams ranged from 2.3 × 10^3^ to 1.7 × 10^6^ GC/100 ml and from 7.6 × 10^2^ to 4.7 × 10^4^ GC/100 ml in river samples. The median values for rivers and streams differed by less than one order of magnitude. Variations in HF183 levels were observed in both beaches ranging from below the limit of quantification to 6.1 × 10^4^ GC/100 ml for Merrion Strand and 2.2 × 10^4^ GC/100 ml for Sandymount Strand. The median concentrations of the marker in marine samples were lower than those of rivers and streams.

All the rivers and streams were continuously impacted by human pollution. In contrast, the ruminant marker was less prevalent in rivers, streams, and bathing waters. Thus, anthropogenic activities are therefore likely to be the primary driver of fecal pollution at the sampling stations in the different watercourses.

### Rivers and Urban Streams Have a Similar ARGs Profile

**FIGURE 4:**
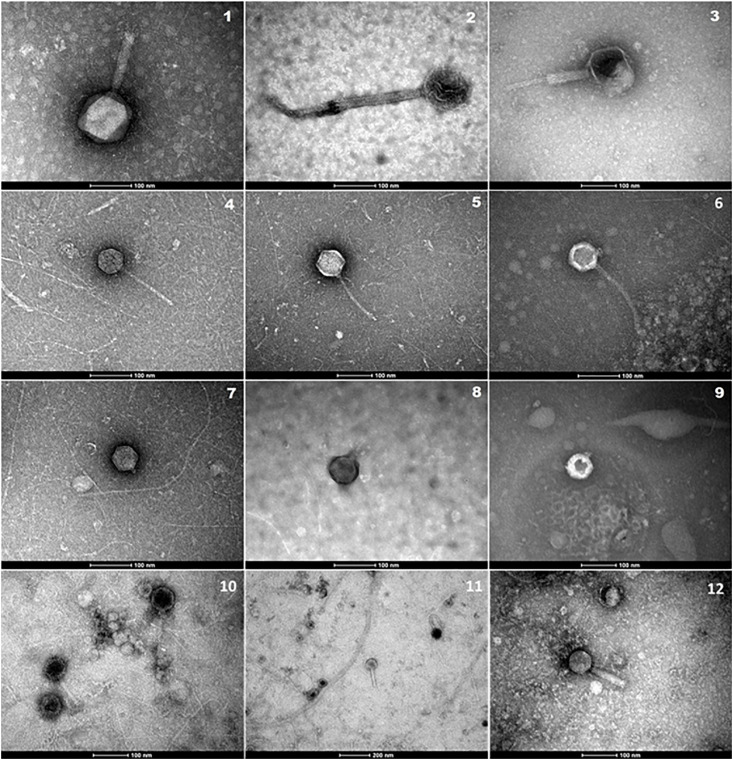
Electron micrographs showing bacteriophages isolated in concentrated water samples from Elm Park Stream (1, 4, 5, 7, 11), River Tolka (2, 8, 10), Trimleston Stream (6, 9), and raw sewage (3, 12). Images 1–3 show *Myoviridae* phages, images 4–6 *Siphoviridae* phages and images 7–9 *Podoviridae* phages. Scale bars represent 100 nm, whereas the scale bar in micrograph-11 represents 200 nm.

Bacteriophages may be an important reservoir of ARGs yet are often overlooked. We therefore wanted to analyze water samples for ARGs in both bacteria and bacteriophages. To validate our bacteriophage enrichment procedure, the concentrate was examined using an electron microscope. Bacteriophages with morphologies corresponding to families *Myoviridae*, *Siphoviridae*, and *Podoviridae* (Demuth et al., 1993; Colomer-Lluch et al., 2011) were present (Figure 4).

**FIGURE 5:**
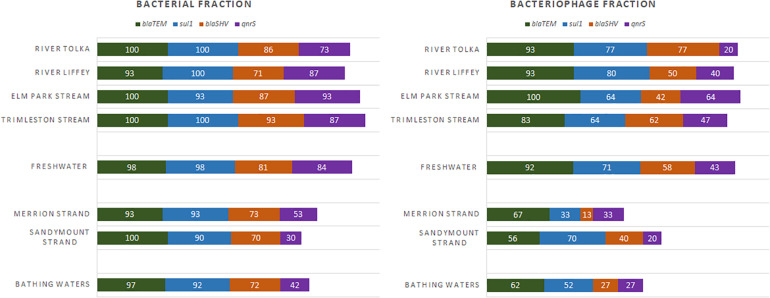
Frequency of detection (%) for the antibiotic resistance genes in freshwater (River Tolka and Liffey, Elm Park, and Trimleston Streams) and bathing waters (Merrion and Sandymount Strands).

**FIGURE 6:**
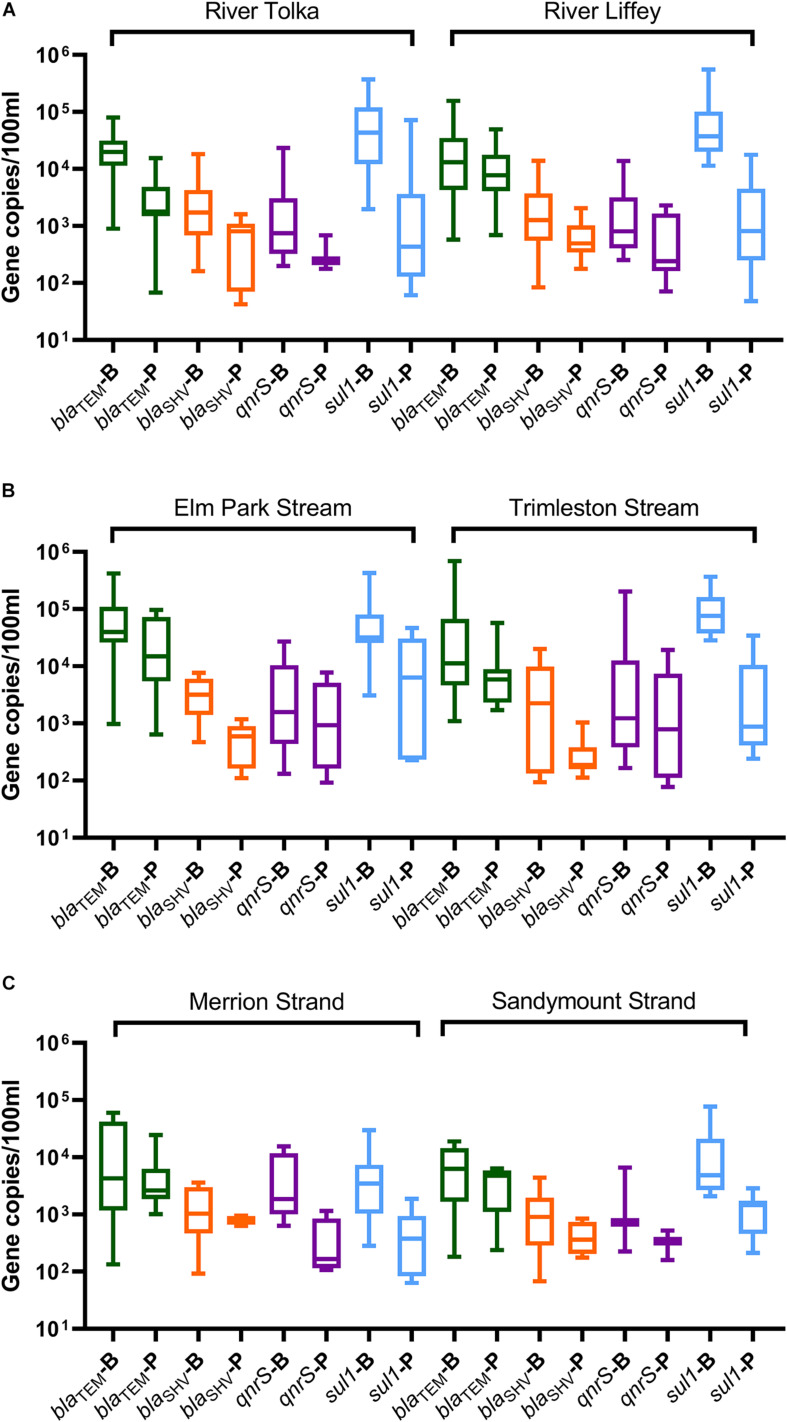
Boxplot representation of the ARGs concentrations in the bacterial (B) and bacteriophage (P) fractions in water samples from **(A)** rivers (Tolka and Liffey), **(B)** streams (Elm Park and Trimleston), and **(C)** bathing waters (Merrion and Sandymount Strands). In the boxplots the lower hinge represents 25% quantile, upper hinge 75% quantile, and center line the median. The whiskers show the maximum and the lowest value. Only values above the quantification limit are plotted.

The presence of antibiotic resistance genes in the bacterial and bacteriophage fractions was assessed by selecting four ARGs, *bla*_*TEM*_, *bla*_*SHV*_, *qnrS*, and *sul1*. Bacterial ARGs were found in 73–100% of the river samples and the percentage of positive samples was even higher in the stream samples (87–100%). However, only 20–93% of the bacteriophage fractions from the rivers and 42–100% from the streams contained ARGs (Figure 5). In all cases the level of ARGs in the bacteriophage fraction was lower than in the bacterial fraction (Figure 6). The bacteriophage DNA preparations did not contain 16S rDNA.

The *bla*_*TEM*_ and *sul1* genes the most prevalent in the bacterial fraction, while *bla*_*TEM*_ was most abundant in the bacteriophage fraction of the river and stream samples. The *bla*_*TEM*_ median levels ranged from 1.1 × 10^4^ to 4.0 × 10^4^ GC/100 ml in the bacterial fraction and from 1.7 × 10^3^ to 1.4 × 10^4^ GC/100 ml in bacteriophage fraction. The *sul1* gene was the most abundant in the bacterial fraction of the River Tolka, River Liffey, and the Trimleston stream with levels ranging from 3.2 × 10^4^ to 7.6 × 10^4^ GC/100 ml. Interestingly, the levels of *sul1* in the bacteriophage DNA were 10- to 100-fold lower.

The median concentrations for the bacterial *bla*_*SHV*_ and *qnrS* genes were in the same range (3 log_10_ GC/100 ml) but were one order of magnitude lower than *bla*_*TEM*_ and *sul1*. For the bacteriophage fraction, the median levels for *bla*_*SHV*_ and *qnrS* genes observed in rivers were in the same range (2 log_10_ GC/100 ml) as those found in the urban streams. However, the *qnrS* gene was more commonly found in the urban streams (47–64%) in comparison to the largest rivers (20–40%). Interestingly, an opposite pattern was obtained for *bla*_*SHV*_. Overall, the profiles of the bacterial ARGs in the urban streams and the largest rivers were similar, since no statistical differences for the majority of ARGs were reported between rivers and streams (Kruskal–Wallis, *p* > 0.05, Supplementary Table 3).

Spearman correlation analysis was performed to establish the relationship between the levels of ARGs and the human fecal marker. Since there was no significant difference between the ARG profiles of rivers and streams, all samples were treated as a single dataset. The *bla*_*TEM*_, *bla*_*SHV*_, and *qnrS* genes in the bacterial DNA fraction correlated moderately with the human fecal marker (Spearman correlation, ρ = 0.292–0.335, *p* < 0.05, Supplementary Table 4). However, there was no significant correlation between the human marker and ARGs in the bacteriophage fraction.

### ARGs in Bathing Waters Have an Urban Profile

The rivers and streams in this study are mostly impacted by human fecal contamination and have the same ARG profile, despite their substantial difference in size. We hypothesized that the discharge of these streams and rivers into Dublin Bay would affect the nearshore marine environment.

All ARGs present in the streams and rivers were also found in the two bathing waters. The detection frequency profile of the ARGs in the rivers, streams, and strands was very similar (Figure 5). The *bla*_*TEM*_ and *sul1* genes were the most frequently detected genes, followed by *bla*_*SHV*_ and *qnrS*. The levels of individual ARGs in both the bacterial and bacteriophage fractions in Merrion and Sandymount Strand did not differ significantly (Mann–Whitney, *p* > 0.05, Supplementary Table 5), which is not surprising, considering that these bathing waters are adjacent to each other. As was observed for the rivers and streams, in the nearshore marine environment *bla*_*TEM*_ and *sul1* were the most abundant ARGs in the bacterial fraction, and *bla*_*TEM*_ was the most abundant in the bacteriophage fraction (Figure 6). The median level of *bla*_*TEM*_ ranged from 2.6 × 10^3^ to 6.3 × 10^3^ GC/100 ml and from 3.7 × 10^2^ to 4.8 × 10^3^ GC/100 ml for *sul1*. As was the case for the rivers and streams, the levels of *bla*_*SHV*_ and *qnrS* were around 0.5 log_10_ lower than the two most abundant genes (Figure 6).

In comparison to the freshwater samples, the median concentration for bacterial ARGs levels (*bla*_*TEM*,_ *bla*_*SHV*_ and *sul1*) decreased by significantly two and ninefold in marine bathing waters (Mann–Whitney, *p* = 0.0001–0.041, Supplementary Table 5 and Supplementary Figure 1), The *qnrS* levels did not change significantly (Mann–Whitney, *p* = 0.466). In contrast, there was no significant reduction in ARG levels for the phage fraction (Mann–Whitney, *p* = 0.093–0.653, Supplementary Table 5 and Supplementary Figure 1).

## Discussion

The rivers Liffey and Tolka rise outside Dublin and flow through an agricultural area before entering the city and discharging into Dublin Bay. In contrast, the two streams included in this study are completely urban and are therefore experiencing different pollution pressures than the much larger rivers. Despite these potential differences in pollution pressures, microbial source tracking analysis showed that the main source of pollution in both rivers and streams in the urban environment is human contamination, which presumably enters the rivers and streams through for example combined sewer overflows and sewerage misconnections.

The four clinically relevant ARGs included in this study *bla*_*TEM*_, *bla*_*SHV*_, *qnrS*, and *sul1*, were present in all four waterways and occurred in both the bacterial as well as in the bacteriophage fraction. These ARGs selected confer resistance to the most common antibiotics prescribed in Ireland (HSE, 2019). Interestingly, the ARG profiles of the four waterways were very similar, both in terms of the levels of ARGs and the frequency of detection. This is consistent with the urban environment as the main fecal impactor on these waterways. In line with this, a moderate level of correlation between the bacterial *bla*_*TEM*_, *bla*_*SHV*_, and *qnrS* and the HF183 marker was observed, but not for *sul1*. In contrast, there was no correlation between the levels of the four ARGs in bacteriophage fraction and HF183. Partial correlation between ARGs and indicators of fecal contamination has been reported previously (Calero-Cáceres et al., 2017).

In the bacterial fraction, the *sul1* and *bla*_*TEM*_ genes were the most prevalent followed by *bla*_*SHV*_ and *qnrS*. The *bla*_*TEM*_ gene was the most prominent ARG in the bacteriophage fraction, followed by *sul1*, *bla*_*SHV*_, and *qnrS*. Resistance to β-lactam antimicrobial agents by *bla*_*TEM*_, *bla*_*SHV*_ are widely distributed in aquatic ecosystems (Colomer-Lluch et al., 2011; Anand et al., 2016; Calero-Cáceres et al., 2017; Zhang et al., 2019), which might indicate that they are particularly amenable to propagation through transduction (Wang et al., 2018a). Sulfonamides are one of the oldest antimicrobial synthetic or semi-synthetic drug classes that have been also used for the treatment of animals (Dasenaki and Thomaidis, 2017) and is an authorized antibiotic for use in aquaculture in Europe (Santos and Ramos, 2018). The *sul1* gene was the second most abundant and variable in concentration in the bacteriophage fraction. This high degree of variability of *sul1* in rivers has also been observed in Mediterranean human-impacted rivers (Calero-Cáceres et al., 2017; Lekunberri et al., 2017) and rivers in China (Yang et al., 2018). Sulfonamide resistance is associated with mobile genetic elements, like class 1 integrons (int1) (Jiang et al., 2019), which may explain the presence in the bacteriophage fraction. Resistance to fluoroquinolones have been associated with clinical *Enterobacteriaceae* isolates as well as in waterborne bacteria, but with lower prevalence in rivers (Poirel et al., 2012; Marti and Balcázar, 2013; Lekunberri et al., 2017).

Aquatic environments are ideally suited for the dispersal of ARGs (Marti et al., 2014a). Rivers and streams may therefore have a lasting effect on the presence of ARGs in the marine environment into which they discharge. In addition, the discharge of wastewater treatment plants will also add to the presence of ARG in the marine environment. The ARG profiles of two bathing waters in Dublin Bay were not significantly different from those of the rivers and streams, although the concentrations and frequency of detection were lower. The *bla*_*TEM*_ gene is also the most abundant in the marine environment, which is consistent with the few marine studies in the Mediterranean Sea and the Indian Ocean, where *bla*_*TEM*_ gene was the most prevalent and abundant gene in the bacteriophage fraction (Calero-Cáceres and Balcázar, 2019; Blanco-Picazo et al., 2020).

Interestingly, there was a significant two to ninefold reduction in median values of the bacterial *bla*_*TEM*_, *bla*_*SHV*_, and *sul1* genes, whereas the levels of *qnrS* did not change significantly. Freshwater or intestinal bacteria carrying ARGs are likely to die off rapidly in the marine environment, which would account for the decrease of three of the four ARGs. Resistance to fluoroquinolone in marine environments is related to the intrinsic resistance of marine *Vibrionaceae* and *Shewanellaceae* family species which possess chromosome-encoded Qnr-like proteins (Poirel et al., 2005; Cattoir et al., 2007). The decrease of bacterial ARGs in the marine environment was not observed for bacteriophage ARGs. In general, decay rates of bacteriophages in seawater are much lower than those of fecally associated bacteria and therefore persist for prolonged periods of time (Mocé-Llivina et al., 2005; Calero-Cáceres and Muniesa, 2016; Wu et al., 2020; Sala-Comorera et al., 2021a).

Once ARGs have been introduced to the phageome, they may be reintroduced into the bacterial metagenome through horizontal gene transfer mechanisms (Muniesa et al., 2013a). Prolonged persistence of bacteriophages carrying ARGs, may result in transfer of these ARGs to marine microbiota, and eventually find their way into human consumers of seafood. In addition, and perhaps more importantly, ARG carrying bacteriophages may accumulate in filter feeders (e.g., oysters) and enter the food chain in this manner. Furthermore, recreational activities taking place in or on the water may expose people to ARG carrying bacteriophages, which once ingested may transfer these ARGs to the microbiota in the intestinal tract. The EU Bathing Water Directive classifies bathing water solely on the presence of *E. coli* and intestinal enterococci, with a view to prevent gastrointestinal and respiratory disease (EU, 2006). Although the use of bacteriophages as additional or alternative indicators for fecal contamination has and is being discussed, the data presented here make a case for the inclusion of bacteriophages as indicators for the potential spread of antimicrobial resistance during recreational use of bathing waters.

## Data Availability Statement

The raw data supporting the conclusions of this article will be made available by the authors, without undue reservation.

## Author Contributions

LS-C: conceptualization, methodology, validation, formal analysis, data curation, writing—original draft, writing—review and editing, and visualization. TN and LR: investigation and validation. AV, LC, NM, JS, and AG: investigation. GO’H and JO’S: writing—review and editing. WM: conceptualization, supervision, writing—review and editing, supervision, project administration, and funding acquisition. All authors contributed to the article and approved the submitted version.

## Conflict of Interest

The authors declare that the research was conducted in the absence of any commercial or financial relationships that could be construed as a potential conflict of interest.

## Publisher’s Note

All claims expressed in this article are solely those of the authors and do not necessarily represent those of their affiliated organizations, or those of the publisher, the editors and the reviewers. Any product that may be evaluated in this article, or claim that may be made by its manufacturer, is not guaranteed or endorsed by the publisher.
