# Supplementary material for: Bacterial and Bacteriophage Antibiotic Resistance in Marine Bathing Waters in Relation to Rivers and Urban Streams
Source: Front Microbiol. 2021 Jul 26;12:718234. doi: 10.3389/fmicb.2021.718234 (PMC8350879; doi:10.3389/fmicb.2021.718234)
Supplement: Supplementary file 1 [file Data_Sheet_1.PDF]

Bacterial and Bacteriophage Antibiotic Resistance in Marine Bathing Waters in Relation to Rivers and Urban Streams

SUPPLEMENTARY MATERIAL

Laura Sala-Comorera[1], Tristan M. Nolan[1], Liam J. Reynolds[1], Anjan Venkatesh[1], Lily Cheung[1], Niamh A. Martin[1], Jayne H. Stephens[1], Aurora Gitto[1], Gregory M. P. O'Hare[2], John J. O'Sullivan[3], Wim G. Meijer[1*].

1. UCD School of Biomolecular and Biomedical Science, UCD Earth Institute and UCD Conway Institute, University College Dublin, Dublin 4, Ireland
2. UCD School of Computer Science and UCD Earth Institute, University College Dublin, Belfield, Dublin 4, Ireland
3. UCD School of Civil Engineering, UCD Dooge Centre for Water Resources Research and UCD Earth Institute, University College Dublin, Dublin 4, Ireland

* Corresponding author: Wim G. Meijer

Tel: (+353) 17162778

Email: wim.meijer@ucd.ie

Key words: antibiotic resistance genes, microbial source tracking, bacteriophages, fecal pollution, rivers, urban streams, bathing waters

**Classification**	**Name**	**Latitude**	**Longitude**
River	Tolka	53.364417	-6.247505
River	Liffey	53.347843	-6.34552
Stream	Elm Park	53.314389	-6.20350
Stream	Trimleston	53.312058	-6.198642
Strand	Merrion	53.316344	-6.203459
Strand	Sandymount	53.325275	-6.206281

Supplementary Table 1. Geographic coordinates of the sampling sites.

**Target Gene**	**Primer sequences**	**Cycling Parameters**	**Quantification (QL) and detection (DL) limit (GC/μl reaction)**	**Reference**
HF183	*F:*ATCATGAGTTCACATGTCCG<br>*R:*TACCCCGCCTACTATCTAATG	45 cycles (95°C - 5s, 60°C - 15s, 72°C - 20s)	2.76	(Seurinck et al., 2005)
CF128	*F:*CCAACYTTCCCGWTACTC<br>*R:*TACCCCGCCTACTATCTAATG	50 cycles (95°C - 15s, 60°C -15s, 72°C - 20s)	10	(Bernhard and Field, 2000)
bla$_{TEM}$	*F:*CTATGGCACCACCAACGATA<br>*R:*ACGGCTTTCTGCCTTAGGTT	40 cycles (95°C - 10s, 60°C -20s, 72°C - 20s)	1.7	(Sidrach-Cardona et al., 2014)
bla$_{SHV}$	*F:*CGCTTTCCCATGATGAGCACCTTT<br>*R:*TCCTGCTGGCGATAGTGGATCTTT	40 cycles (95°C - 15s, 62°C -32s, 72°C - 20s)	1.2	(Xi et al., 2009)

qnrS	*F:*GACGTGCTAACTTGCGTGAT<br>*R:*TGGCATTGTTGGAAACTTG	45 cycles (95°C - 15s, 64°C -30s, 72°C - 20s)	2.4 (QL)<br>1.2 (DL)	(Marti and Balcázar, 2013b)
sul1	*F:*CGCACCGGAAACATCGCTGCAC<br>*R:*TGAAGTTCCGCCGCAAGGCTCG	50 cycles (95°C - 15s, 65°C -30s, 72°C - 30s)	2.4 (QL)<br>1.2 (DL)	(Pei et al., 2006)
16S rRNA	*F:* AGAGTTT-GATCMTGGCTCAG<br>*R:* TACGGYTACCTTGTTACGACTT	35 cycles (94°C - 60s, 55°C -60s, 72°C - 90s) 72 - 90s		(Weisburg et al., 1991)

Supplementary Table 2. Primers, target genes, limit of quantification and detection for the assays and cycling conditions used in this study.

**Kruskal-Wallis test**			
***bla*$_{TEM}$-B**			
P value	0.0565		
Number of groups	4		
Kruskal-Wallis statistic	7.540		
Dunn's multiple comparisons test	Mean rank diff.	Significant	P Value
E bla$_{TEM}$-B vs. *T bla*$_{TEM}$-B	12.07	No	0.3261
E bla$_{TEM}$-B vs. *TK bla*$_{TEM}$-B	13.53	No	0.1856
E bla$_{TEM}$-B vs. *Lbla*$_{TEM}$-B	15.84	No	0.0785
T bla$_{TEM}$-B vs. *TK bla*$_{TEM}$-B	1.467	No	>0.9999
T bla$_{TEM}$-B vs. *Lbla*$_{TEM}$-B	3.771	No	>0.9999
TK bla$_{TEM}$-B vs. *Lbla*$_{TEM}$-B	2.305	No	>0.9999
***bla*$_{TEM}$-P**			
P value	0.0054		
Number of groups	4		
Kruskal-Wallis statistic	12.67		
Dunn's multiple comparisons test	Mean rank diff.	Significant	P Value
E bla$_{TEM}$-P vs. *T bla*$_{TEM}$-P	8.780	No	0.8459

E bla$_{TEM}$-P vs. *TKbla*$_{TEM}$-P	19.42	Yes	0.0041
E bla$_{TEM}$-P vs. *L bla*$_{TEM}$-P	4.878	No	>0.9999
T bla$_{TEM}$-P vs. *TKbla*$_{TEM}$-P	10.63	No	0.4153
T bla$_{TEM}$-P vs. *L bla*$_{TEM}$-P	-3.902	No	>0.9999
TKbla$_{TEM}$-P vs. *L bla*$_{TEM}$-P	-14.54	No	0.0569

***bla*$_{SHV}$-B**

P value	0.7191
Number of groups	4
Kruskal-Wallis statistic	1.342

Dunn's multiple comparisons test	Mean rank diff.	Significant	P Value
E bla$_{SHV}$-B vs. *T bla*$_{SHV}$-B	2.203	No	>0.9999
E bla$_{SHV}$-B vs. *TK bla*$_{SHV}$-B	3.346	No	>0.9999
E bla$_{SHV}$-B vs. *L bla*$_{SHV}$-B	6.846	No	>0.9999
T bla$_{SHV}$-B vs. *TK bla*$_{SHV}$-B	1.143	No	>0.9999
T bla$_{SHV}$-B vs. *L bla*$_{SHV}$-B	4.643	No	>0.9999
TK bla$_{SHV}$-B vs. *L bla*$_{SHV}$-B	3.5	No	>0.9999

***bla*$_{SHV}$-P**

P value	0.4288
Number of groups	4
Kruskal-Wallis statistic	2.768

Dunn's multiple comparisons test	Mean rank diff.	Significant	P Value
E bla$_{SHV}$-P vs. *T bla*$_{SHV}$-P	4.675	No	>0.9999
E bla$_{SHV}$-P vs. *TK bla*$_{SHV}$-P	0.3556	No	>0.9999
E bla$_{SHV}$-P vs. *L bla*$_{SHV}$-P	-2.486	No	>0.9999
T bla$_{SHV}$-P vs. *TK bla*$_{SHV}$-P	-4.319	No	>0.9999
T bla$_{SHV}$-P vs. *L bla*$_{SHV}$-P	-7.161	No	0.6251
TK bla$_{SHV}$-P vs. *L bla*$_{SHV}$-P	-2.841	No	>0.9999

***qnrS*-B**

P value	0.9181
Number of groups	4
Kruskal-Wallis statistic	0.5035

Dunn's multiple comparisons test	Mean rank diff.	Significant	P Value
E qnrS-B vs. *T qnrS*-B	1.544	No	>0.9999
E qnrS-B vs. *TK qnrS*-B	4.11	No	>0.9999
E qnrS-B vs. *L qnrS*-B	2.544	No	>0.9999
T qnrS-B vs. *TK qnrS*-B	2.566	No	>0.9999
T qnrS-B vs. *L qnrS*-B	1	No	>0.9999

TK qnrS-B vs. *L qnrS*-B	-1.566	No	>0.9999
***qnrS*-P**			
P value	0.6937		
Number of groups	4		
Kruskal-Wallis statistic	1.451		
Dunn's multiple comparisons test	Mean rank diff.	Significant	P Value
E qnrS-P vs. *T qnrS*-P	0.04762	No	>0.9999
E qnrS-P vs. *TKqnrS*-P	4.333	No	>0.9999
E qnrS-P vs. *L qnrS*-P	3.333	No	>0.9999
T qnrS-P vs. *TKqnrS*-P	4.286	No	>0.9999
T qnrS-P vs. *L qnrS*-P	3.286	No	>0.9999
TKqnrS-P vs. *L qnrS*-P	-1	No	>0.9999
***sul1*-B**			
P value	0.1615		
Number of groups	4		
Kruskal-Wallis statistic	5.144		
Dunn's multiple comparisons test	Mean rank diff.	Significant	P Value
E sul1-B vs. *T sul1*-B	-11.38	No	0.4474
E sul1-B vs. *TK sul1*-B	-0.7857	No	>0.9999
E sul1-B vs. *L sul1*-B	-0.809	No	>0.9999
T sul1-B vs. *TK sul1*-B	12.17	No	0.3143
T sul1-B vs. *L sul1*-B	11.3	No	0.4295
TK sul1-B vs. *L sul1*-B	-0.8667	No	>0.9999
***sul1*-P**			
P value	0.3381		
Number of groups	4		
Kruskal-Wallis statistic	3.369		
Dunn's multiple comparisons test	Mean rank diff.	Significant	P Value
E sul1-P vs. *T sul1*-P	1.014	No	>0.9999
E sul1-P vs. *TK sul1*-P	8.475	No	0.7025
E sul1-P vs. *L sul1*-P	5.583	No	>0.9999
T sul1-P vs. *TK sul1*-P	7.461	No	0.9262
T sul1-P vs. *L sul1*-P	4.569	No	>0.9999
TK sul1-P vs. *L sul1*-P	-2.892	No	>0.9999

Supplementary Table 3. Summary of Kruskal-Wallis test with Dunn's multiple comparison test for ARGs in rivers and streams.

	bla_{TEM}-B	bla_{TEM}-P	bla_{SHV}-B	bla_{SHV}-P	qnrS-B	qnrS-P	sul1-B	sul1-P
**ρ**	0.308	0.082	0.335	-0.227	0.292	-0.022	0.136	0.129
p-value	0.017	0.570	0.017	0.236	0.038	0.919	0.303	0.434

Supplementary Table 4. Correlations between HF183 and ARGs in rivers and streams.

**Mann Whitney Test**				
		P value	Mann-Whitney U	Significant
Merrion vs Sandymount	*bla*$_{TEM}$-B	0.9314	68	No
Merrion vs Sandymount	*bla*$_{TEM}$-P	0.1645	13	No
Merrion vs Sandymount	*bla*$_{SHV}$-B	0.7424	34	No
Merrion vs Sandymount	*bla*$_{SHV}$-P	0.2667	1	No
Merrion vs Sandymount	*qnrS*-B	0.3758	7	No
Merrion vs Sandymount	*qnrS*-P	>0.9999	5	No
Merrion vs Sandymount	*sul1*-B	0.2766	45	No
Merrion vs Sandymount	*sul1*-P	0.187	11	No
**Mann Whitney Test**				
		P value	Mann-Whitney U	
Freshwater vs Bathing waters	*bla*$_{TEM}$-B	0.0019	403	Yes
Freshwater vs Bathing waters	*bla*$_{TEM}$-P	0.0932	261	No
Freshwater vs Bathing waters	*bla*$_{SHV}$-B	0.0416	297	Yes
Freshwater vs Bathing waters	*bla*$_{SHV}$-P	0.6539	76	No
Freshwater vs Bathing waters	*qnrS*-B	0.4664	240	No
Freshwater vs Bathing waters	*qnrS*-P	0.242	61	No
Freshwater vs Bathing waters	*sul1*-B	<0.0001	129	Yes
Freshwater vs Bathing waters	*sul1*-P	0.3548	209	No

Supplementary Table 5. Summary of paired Mann Whitney test for ARGs between strands and freshwater versus bathing waters.

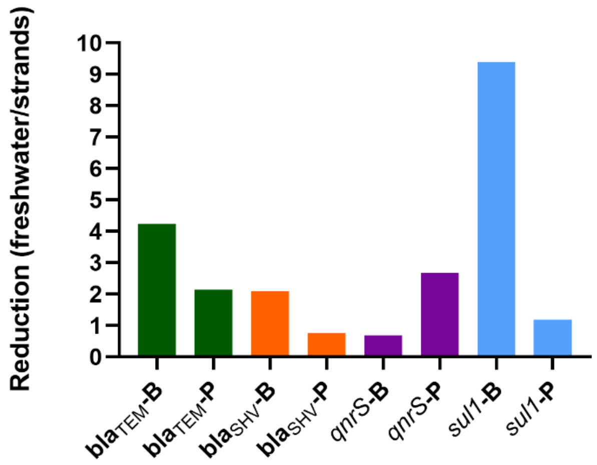

Supplementary Figure 1. Reduction of the median concentration of the freshwater samples with respect to the median concentration of the strands samples.

References

Bernhard, A. E., and Field, K. G. (2000). A PCR assay to discriminate human and ruminant feces on the basis of host differences in Bacteroides-Prevotella genes encoding 16S rRNA. Appl. Environ. Microbiol. 66, 4571–4574. doi:10.1128/AEM.66.10.4571-4574.2000.

Marti, E., and Balcázar, J. L. (2013). Real-time PCR assays for quantification of qnr genes in environmental water samples and chicken feces. Appl. Environ. Microbiol. 79, 1743–1745. doi:10.1128/AEM.03409-12.

Pei, R., Kim, S. C., Carlson, K. H., and Pruden, A. (2006). Effect of River Landscape on the sediment concentrations of antibiotics and corresponding antibiotic resistance genes (ARG). Water Res. 40, 2427–2435. doi:10.1016/j.watres.2006.04.017.

Seurinck, S., Defoirdt, T., Verstraete, W., and Siciliano, S. D. (2005). Detection and quantification of the human-specific HF183 Bacteroides 16S rRNA genetic marker with real-time PCR for assessment of human faecal pollution in freshwater. Environ. Microbiol. 7, 249–259. doi:10.1111/j.1462-2920.2004.00702.x.

Sidrach-Cardona, R., Hijosa-Valsero, M., Marti, E., Balcázar, J. L., and Becares, E. (2014). Prevalence of antibiotic-resistant fecal bacteria in a river impacted by both an antibiotic production plant and urban treated discharges. Sci. Total Environ. 488–489, 220–227. doi:10.1016/j.scitotenv.2014.04.100.

Weisburg, W. G., Barns, S. M., Pelletier, D. A., and Lane, D. J. (1991). 16S ribosomal DNA amplification for phylogenetic study. J. Bacteriol. 173, 697–703.

Xi, C., Zhang, Y., Marrs, C. F., Ye, W., Simon, C., Foxman, B., et al. (2009). Prevalence of Antibiotic Resistance in Drinking Water Treatment and Distribution Systems. Appl. Environ. Microbiol. 75, 5714–5718. doi:10.1128/AEM.00382-09.
